# 

**Published:** 1862-09

**Authors:** 


### CONTENTS.

ORIGINAL ESSAYS AND COMMUNICATIONS.

The Proper Time to Operate. By Dr. W. H. Atkinson........................ 389
Strange Phenomena in the Human Teeth, etc. By W. H. Shadoan .................. 392
The Best Means of Advancing Dental Science. By Dr. J. Allen...................... 394
Plastic Metallic Filling. By Dr. B. Wood............................................ 400

PROCEEDINGS OF SOCIETIES.

American Dental Convention.................................................... 408

EDITORIAL.

American Dental Association................................................... 435

